# Test but not treat: Community members’ experiences with barriers and facilitators to universal antiretroviral therapy uptake in rural KwaZulu-Natal, South Africa

**DOI:** 10.1371/journal.pone.0239513

**Published:** 2020-09-24

**Authors:** Collins Iwuji, Rujeko Samanthia Chimukuche, Thembelihle Zuma, Melanie Plazy, Joseph Larmarange, Joanna Orne-Gliemann, Mark Siedner, Maryam Shahmanesh, Janet Seeley

**Affiliations:** 1 Department of Global Health and Infection, Brighton and Sussex Medical School, University of Sussex, Falmer, United Kingdom; 2 Africa Health Research Institute, Berea, KwaZulu-Natal, South Africa; 3 Univ. Bordeaux, Inserm, Bordeaux Population Health Research Center, Bordeaux, France; 4 Institut de Recherche pour le Développement(IRD), Centre Population et Développement (Ceped), Paris, France; 5 Harvard Medical School, Boston, Massachusetts, United States of America; 6 Institute for Global Health, University College London, London, United Kingdom; 7 London School of Hygiene and Tropical Medicine, London, United Kingdom; Albert Einstein College of Medicine, UNITED STATES

## Abstract

**Introduction:**

Antiretroviral therapy (ART) has revolutionised the care of HIV-positive individuals resulting in marked decreases in morbidity and mortality, and markedly reduced transmission to sexual partners. However, these benefits can only be realised if individuals are aware of their HIV-positive status, initiated and retained on suppressive lifelong ART. Framed using the socio-ecological model, the present study explores factors contributing to poor ART uptake among community members despite high acceptance of HIV-testing within a Treatment as Prevention (TasP) trial. In this paper we identify barriers and facilitators to treatment across different levels of the socio-ecological framework covering individual, community and health system components.

**Methods:**

This research was embedded within a cluster-randomised trial (ClinicalTrials.gov, number NCT01509508) of HIV treatment as Prevention in rural KwaZulu-Natal, South Africa. Data were collected between January 2013 and July 2014 from resident community members. Ten participants contributed to repeat in-depth interviews whilst 42 participants took part in repeat focus group discussions. Data from individual interviews and focus group discussions were triangulated using community walks to give insights into community members’ perception of the barriers and facilitators of ART uptake. We used thematic analysis guided by a socio-ecological framework to analyse participants’ narratives from both individual interviews and focus group discussions.

**Results:**

Barriers and facilitators operating at the individual, community and health system levels influence ART uptake. Stigma was an over-arching barrier, across all three levels and expressed variably as fear of HIV disclosure, concerns about segregated HIV clinical services and negative community religious perceptions. Other barriers were individual (substance misuse, fear of ART side effects), community (alternative health beliefs). Facilitators cited by participants included individual (expectations of improved health and longer life expectancy following ART, single tablet regimens), community (availability of ART in the community through mobile trial facilities) and health system factors (fast and efficient service provided by friendly staff).

**Discussion:**

We identified multiple barriers to achieving universal ART uptake. To enhance uptake in HIV care services, and achieve the full benefits of ART requires interventions that tackle persistent HIV stigma, and offer people with HIV respectful, convenient and efficient services. These interventions require evaluation in appropriately designed studies.

## Introduction

Antiretroviral therapy (ART) provides individual health benefits through a reduction in morbidity and mortality [1, 2] and public health benefits through prevention of HIV transmission [3–5]. These successes coupled with other effective HIV prevention tools [6] make the elimination of HIV possible and highlight the importance of pursuing universal health coverage for all in order to achieve this goal [7]. Although, many mathematical models have shown that it is possible to eliminate HIV with universal testing and immediate ART, these assumptions have been challenging to replicate at the population level even in well-resourced clinical trials [8–10]. One of these trials, the ANRS 12249 TasP trial tested this hypothesis of universal testing and immediate ART in rural KwaZulu-Natal, South Africa, but failed to demonstrate a reduction in HIV incidence [11]. This null finding was largely attributable to poor linkage to care resulting in limited ART uptake observed during the trial [12, 13].

The majority of qualitative studies exploring the factors enhancing and preventing ART uptake and the HIV care continuum were conducted in the era when CD4 thresholds were used to signal eligibility for starting ART [14–17]. These previous studies have shown that expanding ART eligibility can increase ART uptake, but not all patients that had ART-eligible CD4 counts initiated therapy [18]. A systematic review of published data from studies in sub-Saharan Africa looking at retention in HIV care from 2000–2011 showed that approximately one third of eligible patients were lost prior to ART initiation [19]. Despite the increase in ART availability, HIV-related stigma continues to be a barrier to engagement in care [20]. Improved testing, linkage, and ART initiation procedures coupled with stigma reduction interventions are still needed to achieve the UNAIDS 90-90-90 targets to test 90% of people living with HIV (PLHIV), initiate 90% onto treatment and ensure viral suppression of 90% of those people [18, 21].

Studies using qualitative data collection methods investigating factors that facilitate and hinder ART uptake in the test and treat era have been embedded within the public health system. Findings suggest that poor staff attitudes, long waiting times and distance to clinics are barriers to seeking care [22–24]. During the TasP trial, care was provided in purpose-built research clinics, complemented by mobile units in the 22 communities where the trial was implemented. Despite a high community acceptance of home-based HIV testing, only 30% of newly diagnosed individuals linked to care within 6 months of HIV diagnosis with an overall population ART coverage of 51% at the end of the trial [11]. We used qualitative methods to investigate the reasons for this poor uptake of ART, which was a lower proportion than that reported by other test and treat trials nested within traditional health systems [25–27]. We frame our analysis using the socioecological model to show the embedded levels of influence from the individual to the local community and wider health system which affect a person’s interactions with health care, specifically HIV-related care [28, 29]. The socioecological model is described using nested circles that place the individuals at the centre, surrounded in our conceptualisation by the community (the interactions with local services, including traditional healing systems, in the neighbourhood, for example) situated within the wider health system which shapes local health care [30]. Using this model in our analysis highlighted the interactions across the different levels that had an impact on ART uptake by individuals in the TasP trial.

## Methodology

### Setting

The ANRS 12249 TasP trial was a cluster-randomized trial (ClinicalTrials.gov, number NCT01509508) implemented in 22 clusters from March 2012 to June 2016 to investigate the impact of ART regardless of CD4 count on population HIV incidence in the Hlabisa sub-district in rural KwaZulu-Natal [11]. This is a rural setting with scattered homesteads and an estimated HIV prevalence of 30.5% [11]. Migration is high in this setting [31], marriage is uncommon [32] and only 10% of adults are employed [11]. Socio-economic challenges such as a high unemployment rate and high levels of poverty, coupled with sociodemographic factors such as poor educational attainment, high mobility and migration, have contributed to high HIV prevalence [33]. The use of both biomedical and traditional healing systems is common in this setting [29].

The trial protocol has been described previously [12] but, in brief, control arm participants identified as HIV-positive were offered ART according to the South African guidelines (CD4 count ≤350 at trial start [2012], then CD4 count ≤500 from January 2015). Those in the intervention arm were offered ART regardless of CD4 count. Participants living with HIV were referred to dedicated TasP trial clinics within their cluster up to a 45-minute walk for those farthest from their clinic. The qualitative data used in this study came from two control arms and two intervention arms, in which HIV care was provided from mobile clinics attached to mobile/park homes. In addition to providing HIV care, the clinics also catered for other healthcare needs of the participants living with HIV such as management of diabetes, hypertension and other ailments. However, no antenatal care was provided. Participants could opt to receive care from the public primary health care (PHC) clinics where ART would be prescribed according to national guidelines.

### Study design

This study was nested within four of the 22 clusters in the TasP trial from Jan 2013 to July 2014. We used qualitative methods that included focus group discussions (FGDs), in-depth interviews (IDIs) and participant observations, to explore issues related to access to health care, how HIV/AIDS is managed, as well as local practices that facilitate or hinder HIV testing, ART initiation, and adherence in the context of TasP [34]. Using a combination of repeat focus group discussions (FGDs) [35], repeat semi-structured individual interviews (IDIs) with the same participants [36, 37] and participant observations [38] enabled us to determine whether participants’ perceptions or experiences regarding regular and repeat HIV-testing changed or remained the same over time as well as to understand community social norms. Observations from community walks [38] gave more insight into the perceptions and experiences of community members and complemented narratives obtained in IDIs and FGDs.

### Study sample

Overall, we conducted 16 FGDs and 29 IDIs. We purposefully selected individuals for FGDs in four groups, one in each trial cluster to capture diverse interpretations, divided as follows: (i) younger adults (18-35-years, mixed gender, n = 15) who were randomly recruited; (ii) older adults (aged >30, mixed gender, n = 16) randomly recruited with assistance from a local community care giver; (iii) mixed ages (18–65-years, including two traditional health practitioners, n = 11) recruited with assistance from a local community member who worked in a crèche; and (iv) traditional health practitioners (THPs) (aged >35, mixed gender, n = 9) who were recruited using a snowballing technique. The HIV status of these participants was not known to the facilitator, and recruitment was not stratified according to whether individuals were in care in the TasP trial or not. FGDs lasted 45–120 minutes and were conducted in community venues, including school halls.

IDIs were conducted with 20 participants (10 men, 10 women; aged 17–64 years). Ten of the participants were interviewed once, randomly identified by approaching their homesteads in the four trial clusters and interviewed in their homesteads. The other 10 participants were interviewed repeatedly at three time points with (n = 4 males/n = 6 females). Five of the participants were recruited in TasP trial clinics by announcing the existence of the study in a waiting area of the clinic prior to engaging potential participants individually in order to purposefully include participants with a known HIV status. The remaining five were recruited by randomly approaching households across the trial clusters. Repeat interviews were conducted either at the participants’ home or TasP clinic, depending on the participants’ choice and lasted for 30–60 min. All IDIs and FGDs participants were reimbursed an amount of ZAR 50 ($5) for transport and all FGD participants were provided with a lunch pack (juice, fruit and sandwich) after each meeting.

### Data collection

One of the authors (TZ), a social scientist trained in qualitative data collection and a first language *isiZulu* speaker, the local language of the participants, facilitated both interviews and FGDs, audio recorded, transcribed verbatim and translated all data into English. Participants who took part in the FGDs were given cameras during the community walks to capture images of what they considered barriers or facilitators to HIV testing, treatment, adherence and retention in care. The research facilitator did not interfere with the process of capturing images and only asked questions for clarity to capture field notes.

In both IDIs and FGDs, data were collected to understand participants’ perceptions on healthcare services and their utilisation within study communities, including the utilisation of TasP trial clinics, understanding of the TasP concept, local practices to support HIV testing and early ART initiation, and lastly, barriers and facilitators to HIV testing, early ART initiation and adherence.

### Data analysis

Translated data were discussed by the first author with two co-authors (RC, TZ) specifically to understand the dynamic interactions between individual, community and health facility level facilitators and barriers to ART uptake. In this study, we focused our analysis on the individual, community and health system levels of the socioecological model, relevant translated data were extracted manually into an Excel spreadsheet as an initial step to coding. Coded data were repeatedly reviewed by CI, RC and TZ to determine common elements and patterns in the data and to develop thematic categories in line with Braun and Clarkes’ thematic analysis approach [39]. Once a satisfactory map of data was achieved, thematic categories were further defined and refined to identify sub-themes. These were then grouped into individual, community and health system factors. Data from observation notes were used as an additional source to strengthen the analyses. Anonymised quotes from some participants were also included in the manuscript to illustrate certain themes.

### Ethical considerations

The TasP trial was granted ethical approval by the University of KwaZulu-Natal Biomedical Research Ethics Committee (BREC) in 2011 (Ref: BFC 104/11) and received written informed consent from all subjects. Further approval was sought for the full protocol developed for the social science sub-studies, in 2012 (Ref: BE090/12), along with approval from the Community Advisory Board.

## Results

### Characteristics of study participants

Fifty-two participants were enrolled in the study; of whom 36 (69%) were female. The majority (90%) of the participants were unemployed. Fifteen of the participants attained secondary level education, 17 attained primary level education and 20 had not received any formal education. None of the participants attained tertiary level education. The HIV status of all participants in FGDs was unknown to the facilitator. In individual interviews, the HIV status of participants recruited from their homesteads was unknown to the facilitator, unless disclosed by participants during the interview. In repeat individual interviews, one male had an unknown HIV status, one was HIV negative, two were HIV positive and all six females were HIV positive. One female participating in repeat individual interviews relocated from the study area and was not interviewed a third time

### Barriers and facilitators to ART uptake and viral suppression using the socio-ecological model

**Fig 1:**
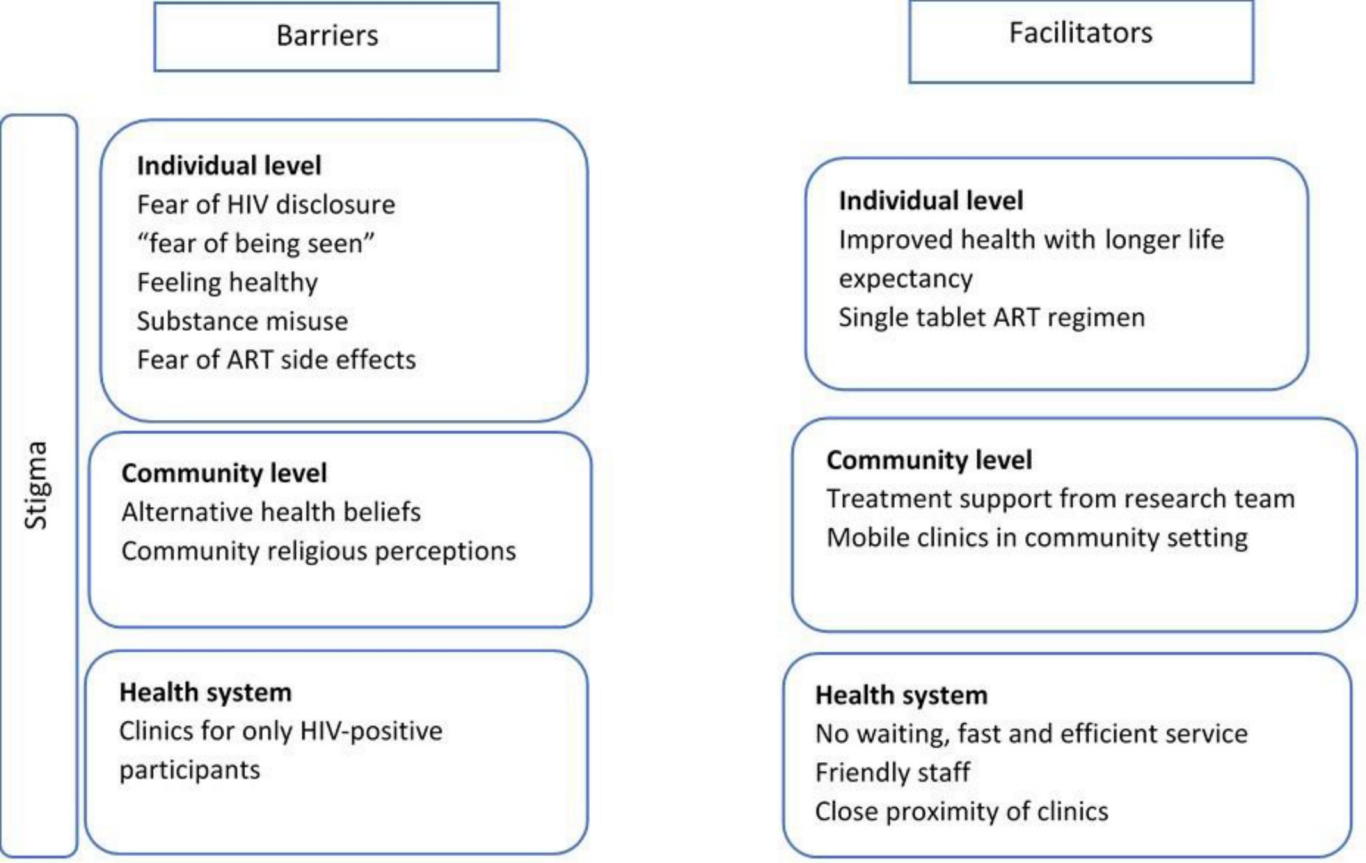
Barriers and facilitators to ART uptake and viral suppression using the socio-ecological model.

Our framing of the data using the socioecological framework to describe the barriers and facilitators to ART uptake at individual, community and health system factors is summarised in the Fig 1 below.

### Barriers

#### Individual level factors

Individuals avoided visiting the trial clinics for ‘fear of being seen’ by other community members whilst accessing HIV services:

*“there are those who are still not keen*. *They have a problem that they will be seen at the park home (Trial clinic) and they say that the park home is full of people who have HIV*. *You see it is something like that*. *You see there are people who go to the clinic not because they are going to check their own illnesses but they keep looking at the people who are going to the research clinic and they say we are even carrying babies who have HIV*. *Now when a lot of people think about that they think if you go to that clinic you are visible*, *they wish they can hide from others*” (Female, 51 years)

Furthermore, some individuals did not see the need to start treatment as they felt healthy and had concerns about ART becoming a problem, perhaps due to potential side-effects. One 46 year-old female commented:

“*They explained side effects that I may experience when I start treatment*. *I took treatment and saw my room moving and I cried out loud thinking I was going to die [laughing]*. *I regretted starting treatment and asked myself why did I do it*? *They told me that it was going to be for a few days*. *It was better after some days and I am no longer having side effects*.”

Another woman of a similar age, was reticent about giving the reasons for interrupting care in early interviews, but in the third and final interview she explained the problems she had faced with side effects:

*Ehhh*, *at the beginning I felt like I was losing my mind*. *…I then stopped taking the treatment and I didn’t go the following month on the date that they had given me*. *I said to myself I am stopping these pills if they are going to make me sick*. *There was a meeting at XX [name of area] and I went to the meeting…*. *They gave me treatment and I continued taking it*. *I don’t have a problem now*.

Other participants taking part in both in-depth interviews and focus group discussions commented that despite people being counselled not to take ART with alcohol, abuse of alcohol was a common problem within the community.

“*My message will be if they can be educated on the harmfulness of alcohol*, *they can understand the importance of their lives*. *Some people will die and leave kids behind just because they preferred drinking alcohol”* (Female, 30 years).

“*This is a photo of a beer [referring to a photo they had taken during the community observation]*. *Most people don’t take good care of themselves*. *Counsellors are educating infected people not to take alcohol with treatment so that treatment can work effectively”* FGD 4 (Youth)

#### Community level factors

Alternative health beliefs are common within Zulu communities and a number of respondents mentioned these beliefs during the interviews. One participant (female, age unknown but above 65) stated that PLHIV default on treatment because traditional healing sources were easily accessible and also needed to treat illnesses they believe to have a spiritual origin and as such utilised frequently as illustrated below:

*“They concentrate on using traditional herbs and forget about getting ART*. *They will continue drinking izichonco (traditional medicine) and stop taking treatment*. *Izichonco (concoction) are these mixtures that people buy*, *and they end up not going to the clinic*.*”*

A 35-year-old traditional healer explained during a focus group discussion that they face challenges in treating PLHIV because they do not disclose their status to them upfront:

“*when someone is ill from this (HIV) they run to us (traditional healers)*, *we have a very big challenge*. *He knows that the illness is eating him away*… *He asks for me to throw my bones so that I can check with the spirits what may be wrong after telling me that he has idliso [an illness caused through bewitchment]*. *He is hiding HIV”*.

A 57-year old male in the second FGD said women go to the clinic but men prefer to use traditional medicine:

*“If we observe things*, *like when you are walking around and observing what is going on around the community*. *You will notice that men do not go to the clinics*, *there are a few men that go to the clinic on the road*, *but you will see many women on the road coming from the clinic and you can see that they are sick*. *Men use traditional medicine more*.*”*

In these communities, health care decisions and choices are influenced considerably by four main sources: social networks, traditional healing knowledge and beliefs, illness perceptions, and illness experiences [29].

Furthermore, community religious beliefs emerged as a barrier to people engaging early in HIV care because they are afraid of being judged when they visited health facilities. One participant during focus group discussion, for example, identified a church during the community walk and said:

*“Churches can be a barrier to test for HIV because you will be regarded as a Christian and not expected to be sexually involved only to find that you are not faithful and have sexual relationships at night*. *You can be infected but will not be able to visit the clinic since you are known as a Christian*” (Male, 21 years).

#### Health system level factors

Some individuals did not like the fact that the TasP clinics only catered for HIV-positive patients:

*“Most people think that you only visit this clinic when you are infected*. *That is what they assume most of the time*. *If you visit this clinic it means that you are infected with HIV” (Female*, *46 years)*.

*“The problem with the research clinic is that it is specific for HIV patients*. *If I visit XY clinic [government clinic] I feel free because I think no one will think that I am going for HIV treatment*. *The problem starts when you have to go to the park homes (Trial clinics) then you will be associated with HIV or TB*. *Someone seeing me going to the park homes will say I am taking HIV treatment although it may happen that I was collecting my child’s treatment” (Female*, *39 years)*.

*“Another challenge*, *like in XY clinic*, *there is a container (park home) [located in government clinic] and its purpose is known*. *There are usually girls sitting in queues and it is difficult for a man to go there because people will be asking themselves what I am doing in an HIV clinic” (Male*, *22 years at FGD 3 (Youth)*.

### Facilitators

#### Individual level factors

Participants reported their motivation to stay healthy and expectation of increased life expectancy as being responsible for their willingness to initiate ART.

*“Yes there is something different*. *When you find out that you are infected and go to the clinic and they give you treatment for HIV*, *in about 2 months to 3 months you recover and see that you are becoming different*, *you see that you are getting better*. *A person can say that I was not like this but now I am looking better because I have started this treatment*. *That person can continue with the treatment and will have testimony for others to say that you see I was not like this but when I started dedicating my life to treatment my life changed” (Male*, *43 years)*

Participants were prescribed a single tablet formulation of antiretroviral therapy. This was said to have facilitated adherence as participants found this easier to take.

“*Also that we now have a place where we can have one pill because we do not have one pill in the big (government) clinics*. *The nurses are also good you can talk to them easily” (Female*, *32 years)*

#### Community level factors

Despite the concerns expressed by respondents, cited above, about the mobile trial clinics being identified as being places where only PLHIV received care, these clinics were viewed very positively by many community members as it made it easier for PLHIV to access treatment because they were close to their homes:

*“This is the Africa Centre (Trial) clinic*. *It is helpful because it is nearby and close in the community*, *it is easy for people to go and collect their treatment*. *If you miss transport to reach the (Government) clinic you will end up not going to the clinic*.*” (Male*, *21 years)*

We observed a paradox that while some people, as noted above, feared the trial clinics as singling out PLHIV, others preferred the model of care in the TasP clinics which catered for only PLHIV to that of the government clinics which catered for both HIV-positive and HIV-negative patients. They were more comfortable in the TasP clinics because all attendees were HIV-positive.

#### Health system level factors

The ‘fast service’ available at the TasP clinics was a big motivation for some which countered the fact that they might be seen by other people receiving care from clinics meant for only PLHIV. Despite this fear of being seen by others, PLHIV found the trial clinics to be a ‘non-judgemental safe place’ as every other person present in the clinic is also HIV-positive.

*“you don’t wait for a long time and you know that people visiting that clinic are all taking this treatment*. *In the government clinic*, *not everyone is taking HIV treatment so they stare at us if we are there for HIV treatment”* (Female, 34 years)

*“The trial clinics are very convenient because it caters for HIV positive people and there are usually no long queues*. *Queues are short and everything is very fast*. *When you go to the research clinic at 8 o’clock*, *by 8*:*30 you are done and going back home*, *but at the government clinic you will only leave at 15h00*. *It is not the same*” (Female, 35 years)

## Discussion

We used the socioecological framework to examine the barriers and facilitators of HIV treatment uptake in a healthcare delivery model that provided access to HIV treatment in HIV care-only trial clinics located in each community. Multiple barriers to treatment uptake were observed at each level of the socioecological framework, mostly attributable to stigma (fear of HIV disclosure, concerns about being seen attending HIV-only treatment facilities and community religious perceptions). This was despite high uptake (92%) of home-based HIV testing [11]. Facilitators of HIV care utilisation included reduced pill burden from single tablet regimens, improved life expectancy on ART and availability of accessible trial clinics in the community offering a fast and efficient service by friendly staff.

Stigma emerged as a dominant over-arching barrier preventing HIV-positive individuals from accessing care for fear of being seen attending HIV-care only clinics. The same observation was made another test and treat trial in South Africa and Zambia [40] where people accessing HIV care in government facilities had to wait in a separate part of the health facility. The research team noted that ‘being seen’ at the clinic was a major barrier to care utilisation [41]. In our own study, despite this perceived barrier, some individuals actually preferred to visit the TasP trial clinics because they found the staff to be friendly, and the services to be quick and efficient. This suggests that the fear of being stigmatised could be overcome for some people by providing more patient-centred care. This can be achieved by bringing care closer to the people with a model of care that is multi-morbidity focused and integrated to address both the concern related to stigma and to geographical accessibility [42, 43]. Transitioning PLHIV as soon as they become stable on ART to adherence clubs or fast-service lane for three to six monthly drug pick-up has also been shown to improve ART uptake and retention in care [44, 45].

Although PLHIV in the TasP communities were complimentary about the proximity of care and shorter waiting times made possible by the trial clinics within the community, the majority of them did not engage with care. The fear of side effects reported by some participants could be related to the historical use of stavudine and zidovudine during the early roll-out of ART in this setting, which many people remembered. Providing information to PLHIV about newer single tablet formulation with fewer side effects could encourage ART initiation and adherence. It could also be that other competing life issues such as livelihood sustenance was prioritised over seeking care [22], especially as ‘feeling healthy’ was mentioned as a reason for delaying care.

Some individuals who delay seeking care or who are in denial of their HIV status often visit traditional healers due to alternative health beliefs [29]. This results in poor uptake of ART and retention in care [46, 47] and consequently increased morbidity and mortality.

Community religious perceptions and the judgemental attitudes of Christians emerged during the community walk as a barrier to accessing HIV services, as people did not want to be seen by their church members seeking care because of the associated connotations of sexual immorality [48]. The church has also been known to influence HIV care in other ways with HIV-positive individuals having discontinued their treatment believing they have been cured of their HIV at their church [49]. Such findings illustrate the influence on individuals not only of local community bodies, such as churches, but also the macro-system which includes societal, religious and cultural values and influences, within which the health-system, community and individual levels of our model are embedded.

Participants identified substance misuse, especially alcohol, as an emerging problem affecting engagement in care in both men and women. Alcohol has been shown to be associated with increased mortality in PLHIV due to poor treatment access and adherence [50].

Our findings suggest that to increase ART uptake interventions would have to address stigma and the time burden of seeking care whilst providing a respectful and efficient service. More attention will need to be paid to substance misuse such as alcohol and recreational drugs and their impact on mental health.

In our study no one intervention meets the need of all PLHIV, rather differentiated multilevel interventions targeting stigma at all levels of the socioecological framework would be required. PLHIV will benefit from the availability of different ART delivery models that they could choose from depending on their changing personal circumstances.

Our research has some limitations. Participants resided in the same community and knew each other. This could have led to social desirability bias in ways we were not able to capture during the interviews. Fewer men participated in the study even though they were most likely not to initiate ART and be retained in care [11, 43, 51]. It is unclear whether additional themes could have emerged if there had been more men in the study. It is also a limitation of our study that we interviewed HIV-positive individuals who linked to care without interviewing those who did not link to care to understand their lived experiences.

We recognise that clinics serving PLHIV might have resulted to unintentional disclosure, however, the study aimed to bring HIV treatment and care services closer to patients and to avoid them being seen in a separate queue at the Government clinic which is the current practice in local primary health care facilities in the study setting. In IDIs, only one individual declared their HIV status as unknown, a larger and more inclusive sample may have enabled a comparison of experiences, attitudes and perceptions of individuals who had tested and knew their status and those who had never tested.

A strength of the study was enabling understanding of HIV-positive individuals behaviour to accessing care in a model of care that shed light on the trade-offs between accessing a fast and efficient service provided by friendly staff and the fear of ‘being seen’ in an HIV-only clinic. This provides a framework to focus interventions to reinforce the facilitators of care observed within a multi-morbidity context that does not single out HIV.

A combination of intervention that addresses the drivers and manifestations of stigma and impact on mental health, in combination with interventions that alleviate the time and economic burden of seeking care such as community access to ART could improve ART uptake and viral suppression in rural South Africa and should be the subject of future research.

## Supporting information

S1 Table Barriers and facilitators -repeat individual in-depth interviews. (PDF)

S2 Table Barriers and facilitators-focus group discussions and individual interviews. (PDF)
