# Supplementary material for: Test but not treat: Community members’ experiences with barriers and facilitators to universal antiretroviral therapy uptake in rural KwaZulu-Natal, South Africa
Source: PLoS One. 2020 Sep 24;15(9):e0239513. doi: 10.1371/journal.pone.0239513 (PMC7514038; doi:10.1371/journal.pone.0239513)
Supplement: S1 Table — (PDF) [file pone.0239513.s001.pdf]

Respondent	Theme	Interview 1	Interview 2	Interview 3
R 9, 34 year old female positive, She has participated in TasP but is not attending the TasP clinic. She is getting her treatment from the Government clinic. She told me that maybe she may have considered moving to the TasP clinic but she had two problems. (1), she was not given a TasP card after she was tested because the counsellors said they had ran out and they were going to come back and give her the card. When they came back, she was busy at work and could not talk to them. They have not been back since. She tested early this year. (2), she fears that the lady working at the clinic as a cleaner will tell other people because she is from the same area of Phondweni where Thobile's family resides. She only disclosed her status to her two sisters and the rest of the family does not know. She told me that her	Value of the TasP Clinic	We were unable to talk but they told me that there is no problem since I am taking my treatment from the other clinic (government clinic) since they work hand in hand.I like to attend the research clinic but I have a problem with one mother who is a cleaner there. She cannot keep to confidentiality and very talkative. I am unable to visit the research clinic and I prefer using the government clinic.	Some people appreciate it since you don't wait a long time like in the government clinic. Some people are not coming to collect treatment there but to check who is taking treatment. It is very nice in the research clinic and don't wait long. Waiting period is usually 3 minutes when there are about 5 patients coming for treatment.The ones I have discussed with are people who have been to the clinic because it was when I had not started using that clinic and my friend suggested that I change from government to research clinic. I was still waiting for the TasP card. They instructed me to take that paper to the clinic. It is very quick there and it is different from arriving in the morning and they start attending to you at around 09h00 then you get home after 14h00. I haven't faced a problem since I started in the research clinic.(No waiting times, fast and efficient)	what I know….i started using the clinic after I talked to you the first time. There are people who came (counsellors) who told me I can use your clinic. When I was using the main clinic I realised that I was wasting my time because you have to wake up very early to go and sit at the clinic until you come back at midday or after that. There are also people who just come to the clinic to watch what other people are doing and when they are going and they laugh about a person who is infected. At the TasP clinic they do the test that they are doing and examine you and give you treatment. When you take cd4 count you see it at the same time, they put some things in your ears too to check you. Everything is good at that clinic

boyfriend tested negative in TasP this year. She does not know how her boyfriend is negative and she is positive. She told me she has never had another partner				
R13, 43 year old male **positive**, He is a TasP participant and has known his status from 2004. He started treatment in 2005; he had TB before he tested for HIV. His children are 13 years old and 9 years old, both girls.	Good Health (Benefits of ART)	I think that now we are on our way to succeed with our health because I have seen a lot of improvement since the availability of HIV treatment. when I look at a lot of people, they were not willing to take treatment. A lot of people ended up being weak and dying because they were not willing to use treatment.	I can say that after 2 to 3 years after starting treatment I noticed improvement from my body.I then decided to stick to treatment for the rest of my life	Yes there is something different. When you find out that you are infected and go to the clinic and they give you treatment for HIV, in about 2 months to 3 months you recover and see that you are becoming different, you see that you are getting better. A person can say that I was not like this but now I am looking better because I have started this treatment. That person can continue with the treatment and will have testimony for others to say that you see I was not like this but when I started dedicating my life to treatment my life changed
R16, **She is on ART** at government clinic since 2008 and she has not seen a reson to leave the NTondweni clinic and join TasP because she has to go to government clinic and vaccinate her children anyway. She was born in kwaShunqa, but moved in with her in-laws in 2002. Her mother is still alive, and her home is not far from her in-laws	Stigma	I didn't tell anyone when I got home because I had seen their behavior. I had seen how they talk if they hear that you have this disease, especially my mother in law. I was quiet about it. My husband was in jail in 2008. [someone at the door]	the Grandmother used to ask me why was I not breastfeeding my baby and I couldn't give her an answer, I kept on ignoring her. She then asked the father when he came back home and he was able to answer her as his mother.He told her that the baby was not being breastfeed because of such reasons that is how she got to know about my status. Even when I go to the clinic to collect my treatment I	I will tell her [laughs] no, it is that most people in the area now know my status. You see when they see this car coming they know that in a certain home someone is taking treatment. One day a friend of my daughter asked me why this car comes here, who is on treatment? I told her I am. She said my father also takes treatment. And I said yes you see, your father is fine, he is well because he is on treatment. I saw that she got okay.

home. She told me that she was encouraged to start treatment when she was told that she was HIV positive because she was pregnant and because she had seen 3 of her uncles dying of HIV and she did not want the same to happen to her.			do tell her that I am going to collect treatment.and she doesn't ask me what that treatment is for.	
R17, 46 year old female, She first found out that she was HIV **positive** last year when she tested in TasP (June 2012). She also started using ART last year June with her cd4 count of 219 and now her cd4 count is above 500. She stated that she would like to be part of the support group. She has not been sexually involved with her partner since she tested last year. She has only disclosed her status to a friend who is also HIV positive, she has not told family members or children.	Fear of side effects (barrier)	I was scared at first but realized that it was not worth it. The clinic was thought to only cater for HIV positive people when it started. There was a meeting held whereby it was explained that the clinic is not only for infected people. The clinic is able to assist TB and diabetes people. I was first scared since it was said to only assist HIV infected people. They will see me going to the clinic and say 'there she goes with HIV' [laughing]. They helped me a lot! They took blood tests after some months of taking treatment and tested my soldiers (CD4 count). They informed me that my soldiers (CD4) have risen up to 500 and something. I then realized the effectiveness of HIV treatment.	It was difficult at first but later I decided to start before getting sick and be noticed by many people that I am sick. Let me just go before they notice. I took a decision and came here. They told me that I would not have any problem. They explained side effects that I may experience when I start treatment. I took treatment and saw my room moving and I cried out loud thinking I was going to die [laughing]. I regretted starting treatment and asked myself why did I do it.They told me that it was going to be for a few days. It was better after some days and I am no longer having side effects.	ehh, I was confused at the beginning but they did explain to me. Ehhh, at the beginning I felt like I was losing my mind. Even the following day I was feeling like my mind was not okay. I then stopped taking the treatment and I didn't go the following month on the date that they had given me. I said to myself I am stopping these pills if they are going to make me sick. They had told me that if I experience any problem I should come back but I didn't go. There was a meeting at government clinic and I went to the meeting. The nurses saw me and they called me I explained what my problem was and then they gave me a date to come back alone because there were many of us there at the time. I went back on the day that they had given me. They gave me treatment and I continued taking it. I don't have a problem now.
