# Supplementary material for: Test but not treat: Community members’ experiences with barriers and facilitators to universal antiretroviral therapy uptake in rural KwaZulu-Natal, South Africa
Source: PLoS One. 2020 Sep 24;15(9):e0239513. doi: 10.1371/journal.pone.0239513 (PMC7514038; doi:10.1371/journal.pone.0239513)
Supplement: S2 Table — (PDF) [file pone.0239513.s002.pdf]

Barriers			
	**Individual**	**Community**	**Health Systems**
**HIV Testing**	INT3,R1: : there are those who are still not keen. They have a problem that they will be seen at the park home and they say that the park home is full of people who have HIV. You see it is something like that. You see there are people who go to the clinic not because they are going to check their own illnesses but they keep looking at the people who are going to the research clinic and they say we are even carrying babies who have HIV. Now when a lot of people think about that they think if you go to that clinic you are visible, they wish they can hide from others. (Individual stigma).	Trial clinic 2- FGD4 , P13:This is a photo of a church. Churches can be a barrier to test for HIV because you will be regarded as a Christian and not expected to be sexually involved only to find that you are not faithful and have sexual relationships at night. You can be infected but will not be able to visit the clinic since you are known as a Christian. (Religious beliefs).	Trial clinic 2 - FGD 4, P13: This photo is a road that represents a long distance. This illustrates that you cannot be able to visit the clinic if you have to walk a long distances. There is no transport available on this road. It is a very long distance from Ndombeni area to Trial clinic 3. This can be a barrier for people to travel such a long distance just for a test. (Long distances to the clinic)
**HIV Treatment**	Trial(TasP) Clinic 1- FGD 4, P1: This next photo is a barrier. I am with a family. Sometimes when you are in a family and they know your status, it may be a barrier for you to engage with health care. Other people become scared to tell their family members that they have a health problem. Others go to the clinic but when they come back they hide and they may even die because they are scared to tell the family, they are scared that the family will stigmatise them. It should not be like that. We should take care of each other in the family. We should be able to give each other advice about what to do. We should not treat that person in a different way and make them have their own spoon and their own plates. I decided to take this photo because one of the barriers in this community can be ones own family members. We need to love each other. (Individual stigma).	Trial clinic 3 - FGD 2, P3: if we observe things, like when you are walking around and observing what is going on around the community. You will notice that men do not go to the clinics, there are a few men that go to the clinic on the road, but you will see a lot of women on the road coming from the clinic and you can see that they are sick. Men use traditional medicine more. (Use of traditional medicine).	Trial clinic 2 - FGD3, P4:There is something about the main clinic (DOH clinic). I think other clinics are better than main clinic. We all know that if you carry a large file, it even has a different colour, it is cream. If you carry that file, we know that something is wrong with you (you are HIV positive). You carry this file from the container (HIV clinic) and back to the main clinic and have to stand in the queue with people looking at you, this is not right . (Lack of privacy at the clinics)

	Trial clinic 2- FGD4,P4: This is a photo of a beer. Most people don't take good care of themselves. Counsellors are educating infected people not to take alcohol with treatment so that treatment can work effectively .<br>My message will be if they can be educated on the harmfulness of alcohol they can understand the importance of their lives. Some people will die and leave kids behind just because they preferred drinking alcohol<br>This is a photo of a cigarrete. People are not educated. It is even written that it causes cancer but people still use it. People are told that if they are on treatment they must stop smoking because it may not be effective when used with cigarrete. Cigarette is capable of destroying your CD4 cells .(substance abuse)		INT1, R15: Yes my concern is that you brought the container to us as patients and we sometimes get sick and need the doctor. Where are we going to get the doctor then?<br>Interviewer: What can make say that you are satisfied with health service you obtain from the container? The full satisfaction will be the availability of the doctor. I knew that I would walk up the cliff and then get to the container. I would walk the same distance to the clinic but have to wait for so much time before being attended to. I still have to wait a long time at the government clinic to collect my BP (blood pressure) treatment .<br>Interviewer: Do you always collect your BP treatment from government the clinic?<br>R 15: Yes. I have been collecting it from the clinic since I started.<br>Interviewer: Are workers from the research clinic aware that you are taking BP treatment?<br>R 15: Yes, they recently phoned me while I was in the government clinic and asked me to visit them to collect my other treatment (HIV treatment). I then went pass research clinic and collected my treatment.<br>Interviewer: Okay,<br>R 15: It then becomes clear that it will take you the whole day. I need to have food to eat whilst in the government clinic whereas I don't bring food with me when visiting the research clinic. I quickly go and collect treatment and go back home and have food.

**Retention**	Trial Clinic 3-FGD2, P3:They concentrate on using traditional herbs and forget about getting ART. They will continue drinking izichonco (traditional medicine) and stop taking treatment . what is izichonco?P7: izichonco are these mixtures that people buy and they end up not going to the clinic (Medical pluralism).	Trial clinic 4 - FGD1, P10: My family does not have a problem with my status if they know that I am infected. The worse is the community. We have been saying that this TasP clinic is only for people who are infected with HIV. Now everyone who is looking at me will say something. They will say I am like this and that and I have a big stomach. They will say she is taking treatment you can see she has a big stomach and we have seen her going into that clinic. That is not good, it makes you feel bad if you are on treatment and you may end up being scared to go to the clinic (Community stigma)	Trial Clinic 3 -3, P13: This is a file for collecting treatment. This file is very good for collecting treatment but it is not good when you are in the government clinic because of stigma. Patients from in the government clinic carry small clinic cards and a person who is HIV positive has to carry this big file and everyone looks at you in a discriminating way because they can tell that you have come to the clinic because you are HIV positive. This file is bad when you are in the government clinic... (Lack of privacy and stigma)
**Facilitators**			
	**Individual**	**Community**	**Health Systems**
**HIV Testing**	Trial Clinic 2-FDG1,P6: I would prefer to be tested at home than visiting the clinic because I may be seen by people from this area at the clinic . You may be seen by other community members? Yes, people can discuss about you. [Group laughing].That is why I prefer to be tested at my home by people that I don't know and who do not know me (individual stigma)	INT 2, R16: Most of the people who have moved from the government clinic to the container (research clinic) are saying that these new clinics are of great assistance. They are able to check you for everything if you are sick. They do all the check-ups. Most of people really benefit from these clinics and I am witnessing that. They are being helped . They say that patients are frequently checked for other diseases from the container clinics (research clinics) whereas other (government) clinics only check your CD4 count or when you are sick and give you some tablets. Patients get regular check-ups from the research clinic. (Mobile clinics)	Trial clinic 4- FGD1, P7:We are not scared and not against this clinic because we have children and you cannot leave this clinic and go to XY government clinic when your child is sick. We like XY government clinic because you can go and have your HIV test without people noticing that. We are scared to come to the research clinic and test for HIV because that is the only service it was made for. If you visit this clinic it becomes obvious that you are HIV infected. They say you have 'that thing ' (Privacy at government clinic)

**HIV Treatment**	INT 3, R13: Yes there is something different. When you find out that you are infected and go to the clinic and they give you treatment for HIV, in about 2 months to 3 months you recover and see that you are becoming different, you see that you are getting better. A person can say that I was not like this but now I am looking better because I have started this treatment. That person can continue with the treatment and will have testimony for others to say that you see I was not like this but when I started dedicating my life to treatment my life changed. (Good health)	INT3,R19: Interviewer: do you think that people who have HIV have enough support?<br>R 19: yes they do a lot.<br>Interviewer: they have support in their families?<br>R 19: yes because others are also encouraged to go and get treatment. Even when a person refuses they persuade him to go until he agrees at other times. You find that he fetches treatment this time but at the second time people in the community start to talk about him and the family encourages him to go back (Good support networks)	Trial clinic 4 - FGD3, P5; I will tell my friend. This clinic is good my child, I will acknowledge that. The government clinic offeres a lot of services, so when a person entres that clinic, no one can actually tell why they are there. But this one (TasP), is still perceived differently. People look at it in a way that scares them. Even at madwaleni clinic, they look at your card to see if you are taking these pills (ARVs) and they look at mine and see that I have never taken this treatment, and then they send me to the door where I can test again. This clinic is helpful (Privacy at goverment clinic)
**Retention**	INT1, R1: I see that taking treatment is a very good thing because your life is safe and you continue living. You become alright. You end up feeling like everyone; it does not mean that you will have a different colour that will show people that you are on treatment, like this and that. But if you do not take treatment, people will see you, you will be visible to people. The end of it is death. But if I am taking it I continue to live and not have a problem .<br>I will start treatment if maybe the research says that I should start, but because I am still walking (feeling well) it should not be treatment that will cause problems for me. You see. If it is treatment that will make me to live and be alright, I will take it and not have a problem . (Good health, Longer life expectancy)	Trial clinic 2 - FGD4, P4: Yes they say they have 'inyongo' and use gum tree for that. This is an Africa Centre vehicle. Africa Centre vehicles are helpful because they visit our homes. The clinic will send these cars to look for you if you are no longer collecting your treatment. These cars will visit you and encourage you to continue taking your treatment. Other people have a problem of disclosing their HIV status to their families and family members can ask questions after these regular visits by Africa Centre. He can then freely take treatment when the family members are aware of his status .(Good support networks TasP team and family)	
